# Spatial Pattern and Spillover of Abatement Effect of Chinese Environmental Protection Tax Law on *PM*_2.5_ Pollution

**DOI:** 10.3390/ijerph19031440

**Published:** 2022-01-27

**Authors:** Fei Han, Junming Li

**Affiliations:** 1 School of Economics, Shanxi University of Finance and Economics, 696 Wucheng Road, Taiyuan 030006, China; Hanfei@sxufe.edu.cn; 2 School of Statistics, Shanxi University of Finance and Economics, 696 Wucheng Road, Taiyuan 030006, China

**Keywords:** environmental protection tax, *PM*_2.5_ pollution, abatement effect, spatial spillover, bayesian statistics, emission inventory

## Abstract

Particulate matter ($PM_{2.5}$) pollution is a threat to public health, and environmental taxation is an important regulatory mode controlling $PM_{2.5}$ pollution. In 2018, China implemented the Environmental Protection Tax Law (EPTL) targeting $PM_{2.5}$ pollution. Based on in-situ monitoring and emission inventory data, a Bayesian hierarchical spatiotemporal model combining a two-period trends difference method was employed to measure the abatement effects of China’s EPTL on $PM_{2.5}$ pollution (AEEPTLPM). On this basis, a spatial spillover index (SSI) of the AEEPTLPM is proposed. Applying this index, we calculated the spatial spillover characteristics of the AEEPTLPM in mainland China at a provincial scale in 2018–2019. The results show that the EPTL has had significant abatement effects on both in-situ-monitored $PM_{2.5}$ concentrations and local total industrial $PM_{2.5}$ emissions. Additionally, the two types of AEEPTLPM display distinct spatial heterogeneity. A correlation between the AEEPTLPM and the degree of $PM_{2.5}$ pollution was observed; areas with serious $PM_{2.5}$ pollution have higher AEEPTLPM levels, and vice versa. The SSI indicates that the AEEPTLPM exhibits significant spatial spillover characteristics, and spatial heterogeneity is also present.

## 1. Introduction

Fine particulate matter ($PM_{2.5}$) poses a serious threat to human health worldwide [1]. In China, with high levels of $PM_{2.5}$ pollution and a large population, the harm is extensive and far-reaching, causing sickness and economic burdens [2]. In response, China launched the Pollution Prevention and Control Battle to control atmospheric pollution by focusing on limiting pollution emissions, adjusting industrial and energy structures, improving policies and regulations, exercising strict supervision and management, and strengthening scientific research, and it has achieved results [3,4,5]. Although annual $PM_{2.5}$ concentrations and the frequency of “heavy haze” events in China have decreased since 2013 [6], in some regions $PM_{2.5}$ pollution remains severe. Premature deaths and a loss of quality of life due to $PM_{2.5}$ totalled approximately 852,000 and 19.98 million people, respectively, in mainland China in 2017. The number of people affected represented 30% of all victims worldwide [7], and the above risks are showing an overall increasing trend in China [8]. However, most of China’s long-term “iron-fisted pollution control” measures are command-and-control environmental regulatory policies. Their effective implementation requires a large investment of human, material, and financial resources, even at the expense of economic development and residents’ quality of life. Without reasonable expectations and market price signals, such measures are ineffective in encouraging polluters to reduce emissions [9]. Although the strict policies have achieved a short-term abatement of $PM_{2.5}$ emissions, a marginal diminishing effect will gradually appear.

As early as 1920, Pigou proposed internalising the external costs of pollution into the production costs of the polluter through government taxation [10]. Pigou’s ideas were adopted by the member states of the Organization for Economic Co-operation and Development. While many scholars have since studied the abatement effects of environmental taxes, a unified view has not been formed. Although few studies have questioned the abatement effects of environmental taxes [11,12], some studies have adopted different methods to demonstrate the emission-reduction effects of different environmental taxes. Consumption taxes such as fuel taxes and vehicle registration taxes can regulate consumers’ consumption behaviour, thereby reducing emissions of pollutants such as $CO_{2}$ and $SO_{2}$. Resource taxes can improve resource utilisation efficiency and reduce pollutant emissions [13,14]. Other taxes for the purpose of pollution prevention and control, such as carbon, garbage, and agricultural pollution taxes, can adjust pollution behaviour and achieve the aim of environmental protection [15,16,17,18].

In 2018, China began to implement the Environmental Protection Tax Law (EPTL). Whether the EPTL can achieve continuous reductions in atmospheric pollutant emissions by effectively guiding polluters to rationally discharge pollutants has attracted widespread concern. Researchers have dedicated in-depth discussion to the significance of the EPTL and related problems in the collection and management of taxes. Dasgupta et al. [19] found that strict environmental taxes were significantly associated with low pollution levels, based on time series analysis. Hettige et al. [20] studied the enterprises of 12 developed and developing countries to discover that strict, effective environmental taxation can reduce sewage discharge. A study by Larsen and Khurshee [21] revealed that the implementation of an environmental tax may improve air quality in the United States. González and Hosoda [22] employed a Bayesian structural time series model and reported that a fuel tax can effectively cut the carbon dioxide emissions of aircrafts. Murray and Rivers [15] researched the effect of a carbon tax in British Columbia to discover that it may reduce greenhouse gas emissions by 5–15%. However, few studies have presented the abatement effects of the EPTL (AEEPTL) on $PM_{2.5}$ pollution (AEEPTLPM). Han and Li [23] explored the AEEPTLPM and annual concentrations in China for 2018. However, as the AEEPTLPM could have temporal hysteresis and spatial spillover, it should be investigated further. Evidence of the spatial spillover effects of the AEEPTLPM is lacking, but a number of studies have researched the spatial spillover of other environmental regulations and $PM_{2.5}$ pollution in urban agglomeration areas of China. Gray and Shadbegian [24] concluded that a company preferred to move production capacity to regions with slack environmental regulations. Zeng and Zhao [25] employed a spatial model to verify the pollution shelter hypothesis. Feng et al. [26] used the spatial Durbin model (SDM) to identify the spatial spillover effects on environmental regulations to $PM_{2.5}$ in 3 urban agglomerations in China. More researchers have focused on the spatial spillovers of $PM_{2.5}$ pollution itself. For example, Shao et al. [27] tested the spatial spillover of $PM_{2.5}$ pollution in China at the province level. Yan et al. [28] used a simple spatial autocorrelation method to analyse the spatial spillovers of $PM_{2.5}$ concentrations in the Beijing-Tianjin-Hebei region in 2016. Other researchers have adopted atmospheric transportation models or econometric models to explore spatial spillovers affected by natural factors, e.g., wind speed and direction [29,30,31,32,33]. Generally, most previous studies have mainly focused on the spatial spillovers of $PM_{2.5}$ pollution itself; those of environmental regulations are less known. Moreover, spatial heterogeneity was not considered in existing studies that examined spatial spillovers.

This study has two targets. The first is to evaluate the average AEEPTLPM in China at the provincial level during 2018–2019 from 2 perspectives: in-situ-monitored $PM_{2.5}$ concentrations and local industrial $PM_{2.5}$ emissions inventories. The other is to establish an index measuring the spatial spillover of the AEEPTLPM under full consideration of spatial heterogeneity and then to calculate the spatial spillover in provinces during 2018–2019.

## 2. Materials and Methods

### 2.1. Materials and Pre-Processing

Four types of data were involved in this study. The first was $PM_{2.5}$ concentration data retrieved through remote sensing. China has monitored air quality in situ since 2013, and only 27% of the 429 prefecture-level cities established in-situ monitoring sites in the first phase. By 2014, the number of prefecture-level cities with ground monitoring stations had increased to 157, accounting for 36.9%. In 2015, 1497 in-situ monitors covered all 429 prefecture-level cities. Table S1 summarises the number of prefecture-level cities in China covered by in-situ monitoring sites in 2013–2019. To improve estimation accuracy for the AEEPTLPM, remote sensing $PM_{2.5}$ concentrations data from 2013 and 2014 were included. The remote sensing $PM_{2.5}$ data, the resolution of which is 0.01°×0.01° (~1 km×1 km), were produced by van Donkelaar’s team [34,35]. The accuracy of the remotely sensed $PM_{2.5}$ annual concentrations at the provincial level for 2015 and 2016 was assessed in this study. The validation results (Table S2) show the root mean square errors in 2015 and 2016 were 1.37 and 1.26 $\mu g/m^{3}$, respectively. The maximums of relative errors were 4.9% and 4.8% in 2015 and 2016, respectively. The absolute and relative errors for the remotely sensed $PM_{2.5}$ annual concentration data in the 31 provincial regions were all under 1.40 $\mu g/m^{3}\;$ and 5.0%. Thus, the validation results certified that the remotely sensed $PM_{2.5}$ annual concentrations from 2013 to 2014 can be integrated with the in-situ monitored data from 2015 to 2018.

The second type of data was in-situ-monitored $PM_{2.5}$ concentrations. Figure S1 shows the spatial distribution of China’s in-situ monitoring sites. To ensure the stability of the results, this paper used the $PM_{2.5}$ concentrations monitored in situ from the 1497 ground sites in mainland China from 1 January 2015 to 31 December 2019, although the number of sites has increased since 2017. The data of $PM_{2.5}$ concentrations corresponding to the 1497 ground sites from 2013 to 2014 were extracted from the remote sensing data. The $PM_{2.5}$ annual concentrations for each provincial region were obtained by zonal average statistics. 

The third type of data was industrial $PM_{2.5}$ emission inventory data at the provincial level. The industrial $PM_{2.5}$ emission inventory data sets were collected from the multi-resolution emission inventory for China (MEIC) produced by Tsinghua University [36,37]. This article uses the latest version (v 1.3) of MEIC data, which contains the total annual $PM_{2.5}$ emissions of the 5 sectors of agriculture, industry, electricity, residence, and transportation. Considering that most objects of the EPTL are industrial enterprises, our study used the total annual $PM_{2.5}$ emissions of the industrial and electric sectors. For convenience, these 2 types of $PM_{2.5}$ emission inventory data are collectively referred to as industrial $PM_{2.5}$ emissions inventory in this paper. As the time range of the latest version (v 1.3) of MEIC data is 2008 and 2010–2017, the provincial annual total industrial $PM_{2.5}$ emissions (PATIPME) from 2018 to 2019 were estimated based on influencing factors. 

The fourth type of data was the influencing factors data of the PATIPME. These data include seven covariates: GDP, the proportion of secondary industry (PSI), disposable income per capita (DIPC), urbanisation rate (UR), total foreign investment (TFI), energy conservation and environmental protection expenditure (ECEPE), and total electricity consumption (TEC). These data were collected from the China Statistical Yearbook and provincial statistical yearbooks.

### 2.2. Estimating the PATIPME of China in 2018 and 2019 

A Bayesian spatiotemporally varying coefficients model (BSTVCM) [38] was used to estimate the PATIPME of China in 2018 and 2019. The process involved two steps. First, the regression coefficients were estimated by the BSTVCM, based on the association between the PATIPME data, $y_{it}$, and the provincial influencing factor variable $X_{it}$ in 2008 and 2010–2017. The specific mathematical expression can be shown as follows:

(1) $$y_{it}\sim N\left(\mu_{it},\sigma_{y}^{2}\right)$$

(2) $$\mu_{it}=\gamma_{i}+b_{0}t+\overset{k}{\underset{i=1}{\sum }}b_{ik}X_{itk}+\overset{K}{\underset{i=1}{\sum }}B_{iK}\ln\left(X_{itK}\right)+\varepsilon_{it}$$

(3) $$\gamma_{i},b_{ik},B_{iK}\sim CAR.Normal\left(adj.S_{y_{i}},adj.S_{n_{i}},adj.S_{W_{i}},\tau_{s}^{2}\right)$$

(4) $$b_{0}\sim Flat\left(-\infty ,\infty \right)$$

where the likelihood distribution of $y_{it}$ is assigned normal distribution; $\mu_{it}$ and $\sigma_{y}^{2}$ are the corresponding expectation and variance; $\gamma_{i}$ is the coefficient of spatial fixed effect; $b_{ik}$ and $B_{iK}$ represent the regression coefficients of the *k*-th and *K*-th corresponding influential variables of the *i*-th provincial area; $X_{itk}$ and $\;\ln\left(X_{itK}\right)$ represent the *k*-th proportional influential variable and *K*-th logarithm influential variable, respectively, of the *i*-th provincial area in the *t*-th year. Considering the spatial structure and non-structural effects, the prior distributions of the coefficients $\gamma_{i},\;b_{ik},B_{iK}$ were assigned using the Besag-York- MOLLIé (BYM) model prior [39]. $b_{0}$ represents the overall trend, and the prior is taken as the non-information prior. $\varepsilon_{it}$ is Gaussian noise.

Second, the PATIPME data of China in 2018 and 2019, $Y_{it}$, can be estimated using the above estimated coefficients and the data for the influencing factors in 2018 and 2019, expressed as follows:

(5) $$Y_{it}=\hat{\gamma_{i}}+{\hat{b}}_{0}t+\overset{k}{\underset{i=1}{\sum }}{\hat{b}}_{ik}X_{itk}+\overset{K}{\underset{i=1}{\sum }}{\hat{B}}_{iK}\ln\left(X_{itK}\right)$$

Seven covariates are included: GDP, PSI, DIPC, UR, TFI, ECEPE, and TEC, of which the four total amount indexes, GDP, TFI, ECEPE, and TEC, are all logarithmic. 

### 2.3. Estimating the AEEPTLPM

#### 2.3.1. The Overall Idea

Previous studies [40,41,42,43] have concluded that the drivers of the annual trends of $PM_{2.5}$ concentrations were dominated by abatements in anthropogenic emissions rather than by meteorological conditions. On 1 January 2018, China began to implement the EPTL, which reduces $PM_{2.5}\;$emissions by regulating the behaviour of polluting enterprises. The Three-year Action Plan for Defending the Blue Sky (TAPDBS) also began to be publicly released on 3 July 2018. Each provincial government (such as Tianjin, Beijing, Chongqing, Henan) drew up implementation plans for the TAPDBS based on their own economic and social development and implemented them successively after September 2018. According to a public report of the Ministry of Ecological Environment, the TAPDBS is a continuation of the Air Pollution Prevention and Control Action Plan (also known as the “Ten Atmosphere”) implemented from 2013 to 2017. In terms of the intensity of regulation, the TAPDBS is stricter than the Ten Atmosphere. Hence, our study assumes that the average annual effect of the TAPDBS implemented from beginning in autumn in 2018 to 2019 is approximate to that of the “Ten Atmosphere” in reducing $PM_{2.5}$ pollution from 2013 to 2017. It should also be noted that, except for the two major administratively ordered environmental regulations, there were other minor administratively ordered environmental regulations. Considering the temporal continuity of these administratively ordered environmental regulations, we assumed that the abatement effects of the primary and other administratively ordered environmental regulations on $PM_{2.5}$ pollution were approximately equal in 2013–2017 and 2013–2019. Under these two assumptions, the extra average annual reduction of $PM_{2.5}$ pollution in 2018–2019 compared with that in 2013–2017 can be regarded as the AEEPTL. Therefore, based on estimation of the local trends of $PM_{2.5}$ concentrations and emissions during 2013–2017 and 2013–2019, the AEEPTL can be measured by 2-period trends difference, and the corresponding mathematical expression is as follows:

(6) $$\Delta AR_{i}=k_{i}^{\left(0\right)}$$

(7) $$\Delta AR\prime_{i}+\Delta EPL_{i}=k_{i}^{\left(1\right)}$$

where $\Delta AR_{i}$ and $\Delta AR\prime_{i}$ represent the average annual effects of the “Ten Atmosphere” from 2013 to 2017 and that of the TAPDBS from 2018 to 2019. According to the above two assumptions, $\Delta AR_{i}$ equals $\Delta AR\prime_{i}$; therefore, by subtracting (6) from (7), the following can be obtained:

(8) $$\Delta EPL_{i}=k_{i}^{\left(1\right)}-k_{i}^{\left(0\right)}$$

where $k_{i}^{\left(0\right)}$ and $k_{i}^{\left(1\right)}$ represent the local trends of $PM_{2.5}$ pollution (monitored concentrations and emissions) in 2013–2017 and 2013–2019, respectively. $\Delta EPL_{i}$ is the average annual AEEPTLPM of the *i*-th provincial region during 2018–2019.

#### 2.3.2. Estimating Local Trends

The Bayesian hierarchical spatiotemporal model (BHSTM) [44] was used to estimate the local trends of provincial annual $PM_{2.5}$ concentrations and PATIPME in China during 2013–2017 and 2013–2019 from the complex spatiotemporal evolution process. The BHSTM is a synthesis of the Bayesian hierarchical model and the spatiotemporal interaction model [44,45] that fully considers spatiotemporal correlations. The mathematical expression is as follows:

(9) $$\rho_{it}^{\left(0\right)}\sim N\left(\gamma_{it}^{\left(0\right)},\sigma_{\rho^{\left(0\right)}}^{2}\right)I\left(0\right)$$

(10) $$\rho_{it}^{\left(1\right)}\sim N\left(\gamma_{it}^{\left(1\right)},\sigma_{\rho^{\left(1\right)}}^{2}\right)I\left(0\right)$$

where $\rho_{it}^{\left(0\right)}$ and $\rho_{it}^{\left(1\right)}$, whose likelihoods are assigned normal distribution, represent the monitored annual $PM_{2.5}$ concentrations and PATIPME of the *i*-th provincial region in the *t*-th year of two time ranges, 2013–2017 and 2013–2019, respectively; $\gamma_{it}^{\left(0\right)}$, $\gamma_{it}^{\left(1\right)}$, $\sigma_{\rho^{\left(0\right)}}^{2}$, $\sigma_{\rho^{\left(1\right)}}^{2}$ are the corresponding means and variances; I(0) means greater than 0; the corresponding spatiotemporal evolution model is expressed as follows:

(11) $$\gamma_{it}^{\left(0\right)}=\alpha^{\left(0\right)}+s_{i}^{\left(0\right)}+\left(K_{0}^{\left(0\right)}t+v_{t}^{\left(0\right)}\right)+k_{1i}^{\left(0\right)}t+\varepsilon_{it}^{\left(0\right)}\;\forall \;t\in \;2013–2017$$

(12) $$\gamma_{it}^{\left(1\right)}=\alpha^{\left(1\right)}+s_{i}^{\left(1\right)}+\left(K_{0}^{\left(1\right)}t+v_{t}^{\left(1\right)}\right)+k_{1i}^{\left(1\right)}t+\varepsilon_{it}^{\left(1\right)}\;\forall \;t\in \;2013–2019$$

where the priors of $\alpha^{\left(0\right)}$ and $\alpha^{\left(1\right)}$ are assigned with non-information priors, representing the general fixed effects; $s_{i}^{\left(0\right)}$ and $s_{i}^{\left(1\right)}$ represent the overall spatial relative risk of $PM_{2.5}$ pollution during the two periods; $\left(K_{0}^{\left(0\right)}t+v_{t}^{\left(0\right)}\right)$ and $\left(K_{0}^{\left(1\right)}t+v_{t}^{\left(1\right)}\right)$ describe the overall trend of $PM_{2.5}$ pollution in 2013–2017 and 2013–2019; $v_{t}^{\left(0\right)}$ and $v_{t}^{\left(1\right)}$ describe the nonlinear overall trends. The priors of $s_{i}^{\left(0\right)}$, $s_{i}^{\left(1\right)}$, $k_{1i}^{\left(0\right)}$, $k_{1i}^{\left(1\right)}$ adopt the BYM model prior [35], and $v_{t}^{\left(0\right)}$ and $v_{t}^{\left(1\right)}$ adopt BYM model priors in the temporal dimension. $\varepsilon_{it}^{\left(0\right)}$ and $\varepsilon_{it}^{\left(1\right)}$ represent the corresponding Gaussian errors, where the priors are allocated by the Gaussian distribution. The priors of 1/$\sigma_{p\left(0\right)}^{2}$, 1/$\sigma_{p\left(1\right)}^{2}$, 1/$\tau_{s}^{2}$, 1/$\tau_{T}^{2}$, 1/$\sigma_{\varepsilon }^{2}$ adopt the gamma distribution. The related Bayesian statistics estimations in this study were implemented by WinBUGS 14.0.

### 2.4. A Spatial Spillover Index of the AEEPTLPM 

Due to atmospheric flow, the monitored $PM_{2.5}$ concentrations are the synthetic observation results integrating $PM_{2.5}$ emissions from local and surrounding areas. However, the PATIPME reflect $PM_{2.5}$ emissions from local industrial enterprises. To measure the spatial spillover of the AEEPTLPM, we established a spatial spillover index (SSI) of the AEEPTLPM. The mathematical form is as follows:

(13) $$\emptyset_{i}=\frac{E_{i}^{\left(GM\right)}/WE_{i}^{\left(GM\right)}}{\left(\frac{1}{\alpha_{i}}\cdot E_{i}^{\left(EI\right)}\right)/\left(\frac{1}{\beta_{i}}\cdot WE_{i}^{\left(EI\right)}\right)}$$

where $E_{i}^{\left(EI\right)}$ and $WE_{i}^{\left(EI\right)}$ represent the AEEPTL on industrial $PM_{2.5}$ emissions of the *i*-th provincial region and its surrounding regions; the parameters $\alpha_{i}$ and $\beta_{i}$ represent the proportions of the AEEPTL on industrial $PM_{2.5}$ emissions in the AEEPTL on the total $PM_{2.5}$ emissions (including five sectors) in the *i*-th provincial region and its surrounding regions. $E_{i}^{\left(GM\right)}$ and $WE_{i}^{\left(GM\right)}$ represent the AEEPTL on in-situ-monitored $PM_{2.5}\;$ concentrations of the *i*-th provincial region and its surrounding regions. $WE_{i}^{\left(EI\right)}$ and $WE_{i}^{\left(GM\right)}$ are, respectively, calculated on average from $E_{i}^{\left(EI\right)}$ and $E_{i}^{\left(GM\right)}\;$ of the spatial adjacency areas of the *i*-th provincial region. The criterion of the spatial neighbourhood adopted a combination with a maximum distance of 750 km and “Queen contiguity”, including common borders and vertices. This combined criterion can rationally extend the spatial adjacency range; for example, Hainan, an island province, has three spatial neighbourhood provinces.

If there exist no spatial spillovers in the *i*-th provincial area and its surrounding regions, the in-situ-monitored $PM_{2.5}$ concentrations in this region and its surrounding regions are completely determined by the corresponding total $PM_{2.5}$ emissions. Under this assumption, the ratio between $E_{i}^{\left(GM\right)}$ and $WE_{i}^{\left(GM\right)}$ is equal to that between $\left(\frac{1}{\alpha_{i}}\cdot E_{i}^{\left(EI\right)}\right)$ and $\left(\frac{1}{\beta_{i}}\cdot WE_{i}^{\left(EI\right)}\right)$, that is, ∅=1. 

If spatial flow exists, two scenarios will arise for one provincial region. The first scenario is local $PM_{2.5}$ pollutants overflowing into surrounding regions due to atmospheric circulation. The $PM_{2.5}$ annual concentrations caused by $PM_{2.5}$ emissions in the *i*-th provincial region in *t* year are denoted as $PM_{it}$, and the decreased value of $PM_{2.5}$ annual concentrations of this region caused by spatial overflowing into its surrounding areas is denoted as $pm_{it}$. Then, the monitored $PM_{2.5}$ annual concentrations in this region can be expressed as follows:

(14) $$PM\prime_{it}=PM_{it}-pm_{it}$$

Similarly, the monitored $PM_{2.5}$ annual concentrations in the *i*-th provincial area in the *t* − 1 year can be expressed as

(15) $$PM\prime_{it-1}=PM_{it-1}-pm_{it-1}$$

Subtracting (15) from (14), the AEEPTL on the monitored $\;PM_{2.5}$ annual concentrations of the *i*-th area can be expressed as follows:

(16) $$E_{i}^{\left(GM\right)\prime }=E_{i}^{\left(GM\right)}-\Delta E_{i}^{\left(GM\right)}$$

Similarly, the monitored $PM_{2.5}$ annual concentrations in the surrounding areas of the *i*-th provincial region in the *t* and *t* − 1 year, $WPM\prime_{it}$ and $WPM\prime_{it-1}$, can be expressed as

(17) $$WPM\prime_{it}=WPM_{it}+pm_{it}$$

(18) $$WPM\prime_{it-1}=WPM_{it-1}+pm_{it-1}$$

Subtracting (18) from (17) yields

(19) $$WE_{i}^{\left(GM\right)\prime }=WE_{i}^{\left(GM\right)}+\Delta E_{i}^{\left(GM\right)}$$

Combining formula (13) results in the following:

(20) $$\emptyset_{i}=\frac{E_{i}^{\left(GM\right)\prime }/WE_{i}^{\left(GM\right)\prime }}{\left(\frac{1}{\alpha_{i}}\cdot E_{i}^{\left(EI\right)}\right)/\left(\frac{1}{\beta_{i}}\cdot WE_{i}^{\left(EI\right)}\right)}$$

Substituting (16) and (19) into (20) gives

(21) $$\emptyset_{i}=\frac{\left(E_{i}^{\left(GM\right)}-\Delta E_{i}^{\left(GM\right)}\right)/\left(WE_{i}^{\left(GM\right)}+\Delta E_{i}^{\left(GM\right)}\right)}{\left(\frac{1}{\alpha_{i}}\cdot E_{i}^{\left(EI\right)}\right)/\left(\frac{1}{\beta_{i}}\cdot WE_{i}^{\left(EI\right)}\right)}$$

Since $\Delta E_{i}^{\left(GM\right)}>0$, therefore,

(22) $$\frac{E_{i}^{\left(GM\right)}-\Delta E_{i}^{\left(GM\right)}}{WE_{i}^{\left(GM\right)}+\Delta E_{i}^{\left(GM\right)}}<\frac{\frac{1}{\alpha_{i}}\cdot E_{i}^{\left(GM\right)}}{\frac{1}{\beta_{i}}\cdot WE_{i}^{\left(GM\right)}}$$

Combining (21) and (22) yields

(23) $$\emptyset_{i}<1$$

This means the spatial spillover phenomena occurred in the *i*-th provincial region if the corresponding $\emptyset_{i}<1$.

The second scenario is $PM_{2.5}$ pollutants emitted from surrounding regions overflowing into the local area due to atmospheric circulation. Based on the similar mathematical derivation, the following can be obtained:

(24) $$\emptyset_{i}=\frac{\left(E_{i}^{\left(GM\right)}+\Delta WE_{i}^{\left(GM\right)}\right)/\left(WE_{i}^{\left(GM\right)}-\Delta WE_{i}^{\left(GM\right)}\right)}{\left(\frac{1}{\alpha_{i}}\cdot E_{i}^{\left(EI\right)}\right)/\left(\frac{1}{\beta_{i}}\cdot WE_{i}^{\left(EI\right)}\right)}$$

Since $\Delta WE_{i}^{\left(GM\right)}>0$, therefore,

(25) $$\frac{E_{i}^{\left(GM\right)}+\Delta WE_{i}^{\left(GM\right)}}{WE_{i}^{\left(GM\right)}-\Delta WE_{i}^{\left(GM\right)}}>\frac{\frac{1}{\alpha_{i}}\cdot E_{i}^{\left(GM\right)}}{\frac{1}{\beta_{i}}\cdot WE_{i}^{\left(GM\right)}}$$

and

(26) $$\emptyset_{i}=\frac{\left(E_{i}^{\left(GM\right)}+\Delta WE_{i}^{\left(GM\right)}\right)/\left(WE_{i}^{\left(GM\right)}-\Delta WE_{i}^{\left(GM\right)}\right)}{\left(\frac{1}{\alpha_{i}}\cdot E_{i}^{\left(EI\right)}\right)/\left(\frac{1}{\beta_{i}}\cdot WE_{i}^{\left(EI\right)}\right)}>1$$

In summary, the value ranges of $\emptyset_{i}$ indicate three scenarios of spatial spillover: $\emptyset_{i}=1$, no spatial spillover; $\emptyset_{i}<1$, local $PM_{2.5}$ pollutants overflowing into surrounding regions; $\emptyset_{i}>1$, $PM_{2.5}$ pollutants emitted from surrounding regions overflowing into the local area.

## 3. Results

### 3.1. AEEPTL on In-Situ-Monitored PM_2__.__5_ Concentrations

As mentioned, the AEEPTL on in-situ-monitored $PM_{2.5}$ concentrations were estimated based on the corresponding local trends, $k_{1i}^{\left(0\right)}$ and $k_{1i}^{\left(1\right)}$, in 2 stages, 2013–2017 and 2013–2019. Figures S2 and S3 illustrate the posterior median estimations of the local trends of in-situ monitored $PM_{2.5}$ annual concentrations in 2013–2017 and 2013–2019, by the BHSTM. Subsequently, the AEEPTL on in-situ-monitored $PM_{2.5}$ concentrations may be obtained through making difference between the 2 local trends in the 2 phases, $k_{1i}^{\left(1\right)}-k_{1i}^{\left(0\right)}$.

**Figure 1:**
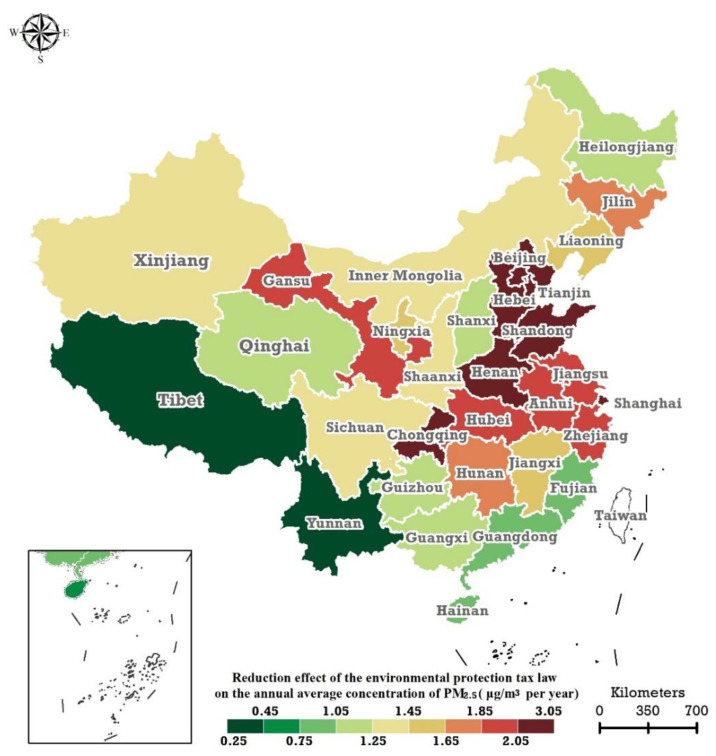
Spatial distribution of the abatement effects of the Environmental Protection Tax Law on the in-situ monitored $PM_{2.5}$ annual concentrations of the 31 provincial regions of the Chinese mainland in 2018–2019.

Figure 1 shows the spatial distribution of the AEEPTL on monitored annual $\;PM_{2.5}$ concentrations at the provincial level in mainland China during 2018–2019. The spatial patterns of the AEEPTL on the monitored $PM_{2.5}$ annual concentrations have significant spatial heterogeneity. Specifically, the foremost seven AEEPTL levels of monitored $PM_{2.5}$ concentrations were observed in Beijing, Hebei, Tianjin, Shandong, Henan, Chongqing, and Shanghai; the corresponding values are –3.05, –2.40, –2.71, –2.33, –2.18, –2.12, and –2.13 $\mu g/m^{3}$ per year, respectively. The AEEPTL on $PM_{2.5}$ concentrations in the southern coastal, western, and southwestern regions were lower, and the lowest values occurred in Hainan, Yunnan, and Tibet, with corresponding values of –0.80, –0.38, and –0.25 $\mu g/m^{3}$ per year, respectively. The AEEPTL on $PM_{2.5}$ concentrations of Inner Mongolia, Shaanxi, and Sichuan were at a moderate level. 

### 3.2. AEEPTL on Local Industrial PM_2__.__5_ Emissions 

#### 3.2.1. Estimation Accuracy of the PATIPME

**Figure 2:**
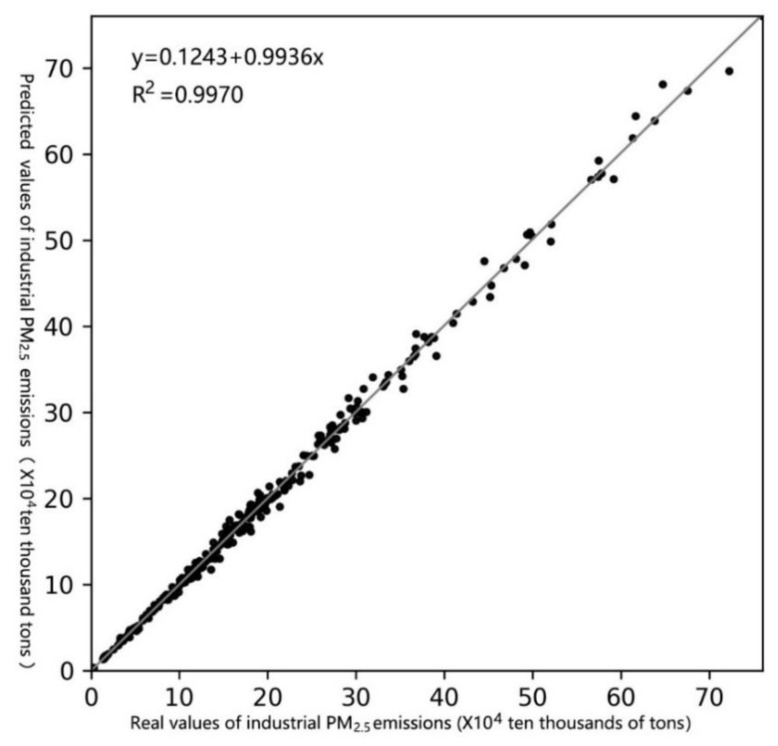
Scatters and fitting line of real and estimated industrial annual total $PM_{2.5}$ emissions of the 31 provincial regions in mainland China from 2013 to 2017.

Table 1 lists the estimated relative errors of the PATIPME in mainland China from 2013 to 2017. The maximum and minimum were 0.2% and 13.1%. The estimated root mean square error (RMSE) of each province from 2013 to 2017 was less than 10%; the maximum RMSE for the 31 provinces was 9.5%; the overall RMSE was 5%. Figure 2 illustrates the scatters and fitting line of the real and estimated PATIPME from 2013 to 2017. It can be seen that the estimation and real values are closely distributed on the diagonal. The fitting line is also highly coincident with the diagonal. Its corresponding fitted slope is 0.9936, which is very close to 1, and the fitting $R^{2}$ is as high as 0.9970. The results indicate that the BSTVCM can obtain high accuracy in estimating the PATIPME of mainland China. 

#### 3.2.2. Estimation of the AEEPTL on Local Industrial $PM_{2.5}$ Emissions

Based on the PATIPME collected from 2013 to 2017 and the PATIPME estimated for 2018 to 2019, the AEEPTL on industrial PM2.5 emissions was assessed. Before estimating the AEEPTL on industrial $PM_{2.5}$ emissions, the 2 local trends or annual changes in the PATIPME in two stages, 2013–2017 and 2013–2019, had to be calculated by the BHSTM. The results of the annual changes in the PATIPME in the 2 periods are shown in Figures S4 and S5. The annual change from 2013–2019, $k_{1i}^{\left(1\right)}$, subtracting measures for 2013–2017, $k_{1i}^{\left(0\right)}$, can generate the AEEPTL on the PATIPME in mainland China at the provincial level during 2018–2019.

**Figure 3:**
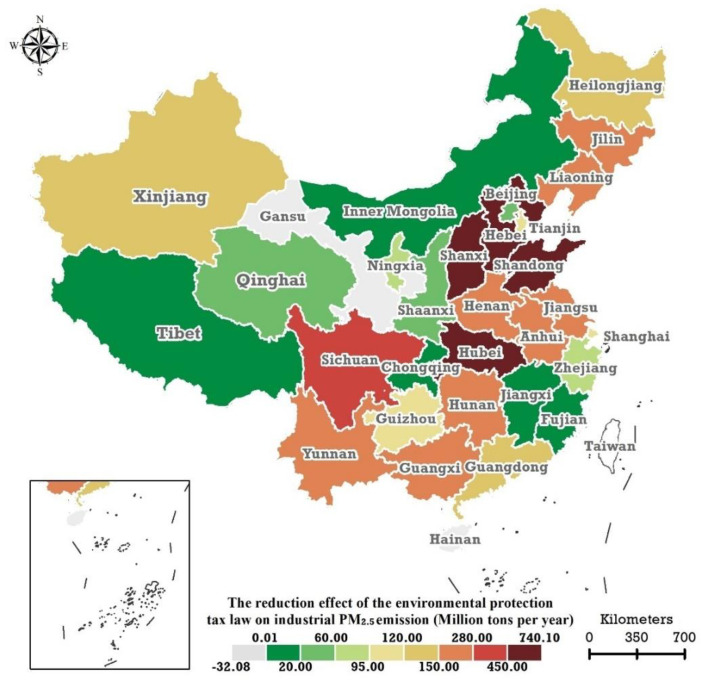
Spatial distribution of the abatement effects of the Environmental Protection Tax Law on the annual total industrial $PM_{2.5}$ emissions of the 31 provincial regions in the Chinese mainland in 2018–2019.

The spatial distribution of the AEEPTL on the PATIPME in mainland China in 2018–2019 is illustrated in Figure 3. In general, the highest level of the AEEPTL on the PATIPME occurred in the southeast region of China, in Hebei, Shanxi, Shandong, and Hubei, with corresponding values of −740.10, −693.60, −598.60, and −457.80 million tonnes per year. Beijing, Zhejiang, Jiangxi, Fujian, and Chongqing had lower AEEPTL levels, with corresponding values of −44.53, −94.03, −10.44, −7.90, and −5.97 million tonnes per year. Except for Xinjiang, five provincial regions (Tibet, Qinghai, Inner Mongolia, Ningxia, and Shaanxi) located in western China had lower levels of AEEPTL on the PATIPME, at −2.63, −21.7, −4.58, −62.63, and −38.71 million tonnes per year, respectively.

The 10 provincial regions of Hebei, Shanxi, Shandong, Hubei, Sichuan, Jilin, Jiangsu, Hunan, Guangxi, and Yunnan experienced the highest AEEPTL on the PATIPME (red series in Figure 3). However, the 10 provinces with the highest PATIPME were Shandong, Henan, Anhui, Guangdong, Hebei, Shanxi, Jiangsu, Inner Mongolia, Liaoning, and Guangxi. By comparison, five (Henan, Anhui, Guangdong, Inner Mongolia, and Liaoning) were among the ten provinces with the highest industrial $PM_{2.5}$ emissions but not the ten provinces with the highest AEEPTL on industrial $PM_{2.5}$ emissions. 

### 3.3. SSI of the AEEPTLPM

**Figure 4:**
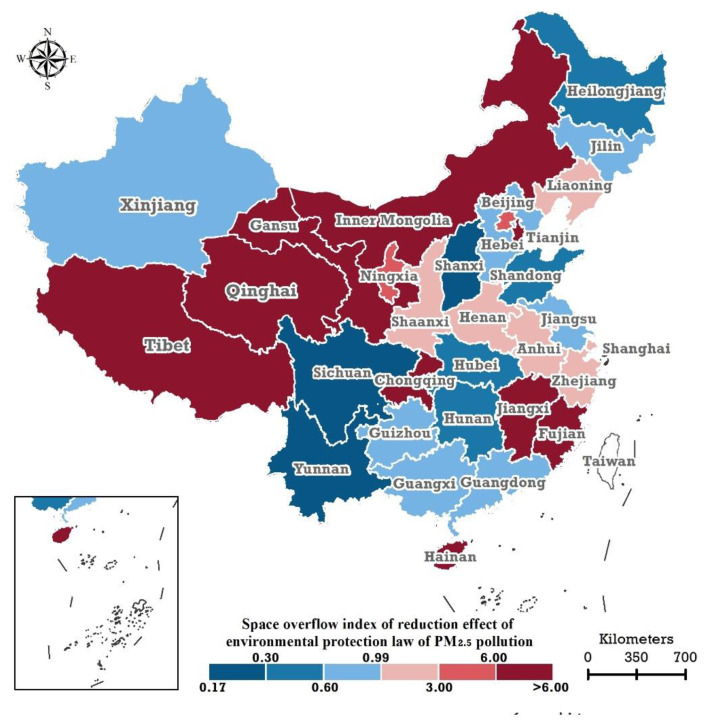
Spatial distribution of the spatial spillover index of the abatement effects of the Environmental Protection Tax Law on $PM_{2.5}$ pollution in the 31 provincial regions of the Chinese mainland.

According to the SSI proposed in this study, we calculated the SSI of the AEEPTLPM in China at the provincial scale from 2018–2019 (Table 2). It should be noted that 2 provinces, Hainan, and Gansu, had no AEEPTL on industrial $PM_{2.5}$ emissions but had AEEPTL on in-situ-monitored annual $PM_{2.5}$ concentrations. According to the idea of constructing the SSI, the corresponding SSIs for these two provinces should be larger, so the SSIs of Gansu and Hainan were classified as at the highest level (dark red in Figure 4).

Specifically, an SSI of less than 1.0 was observed for 14 provincial regions (blue in Figure 4), namely Shanxi, Yunnan, Sichuan, Hubei, Guangxi, Shandong, Hebei, Hunan, Xinjiang, Jilin, Guangdong, Jiangsu, Guizhou, and Heilongjiang, where the minimum and maximum are 0.21 and 0.99. There are 17 provincial regions where the SSIs were greater than 1.0 (red in Figure 4): Liaoning, Henan, Zhejiang, Anhui, Ningxia, Shanghai, Shaanxi, Qinghai, Tianjin, Tibet, Fujian, Beijing, Jiangxi, Inner Mongolia, Chongqing, Gansu, and Hainan, with a minimum of 1.26. Excepting Gansu and Hainan, the 3 highest SSIs occurred in Jiangxi (23.63), Inner Mongolia (28.11), and Chongqing (56.26), and the lowest three occurred in Liaoning (1.26), Anhui (1.51), and Henan (1.57).

In recent years, Beijing and Tianjin have been vigorously controlling atmospheric pollution, and many industrial enterprises have moved to the surrounding areas (mainly Hebei Province). As a result, the locally emitted $PM_{2.5}$ pollutants in both places have been significantly reduced, but, due to natural conditions such as terrain, the AEEPTLPM of the two provinces is greatly affected by the spatial spillover of atmospheric circulation. This study has also empirically proved that the corresponding SSIs of Beijing and Tianjin were greater than 1.0. Regarding Sichuan and Chongqing, the former is located on the west and the latter on the east of the Sichuan Basin. Due to the prevailing northwest wind in winter, part of the $PM_{2.5}$ pollutants emitted in Sichuan naturally overflow into Chongqing. Therefore, the SSI of Sichuan was less than 1.0; correspondingly, the SSI of Chongqing was greater than 1.0. The SSIs for Henan, Anhui, Jiangxi, and Fujian were greater than 1.0. We suspect that these 4 provinces would be affected by the spatial spillover of $PM_{2.5}$ emissions from their surrounding areas also due to the prevailing northwest wind in winter. For Hainan, its industrial structure is dominated by tertiary industries, such as tourism, and its local emissions of $PM_{2.5}$ pollutants are relatively low. However, because Hainan has an island terrain with the lowest altitude, it is vulnerable to the spatial spillover of $PM_{2.5}$ pollution in the northern region.

## 4. Discussion

Based on in-situ monitoring data and emission inventory data, this study proved that the EPTL has had reduction effects on the in-situ-monitored $PM_{2.5}$ concentrations and on local industrial $PM_{2.5}$ emissions. The statistical results produced by this study fully affirm the importance of the EPTL in improving air quality and provide evidence for further deepening China’s green tax reform against the background of carbon neutrality. China has long used pollutant discharge fees to control pollution. However, due to factors such as their size and lack of enforcement, the abatement effect of pollutant discharge fees is not significant. The EPTL was implemented in 2018. It adopts a taxation design of “paying more for more emissions, paying less for fewer emissions, and paying nothing for no emissions” to restrict emissions behaviour and reduce the emissions of polluting enterprises. In practice, the EPTL is deemed only a translation of the pollution discharge fee system, and its abatement effects have been questioned. However, the results of this paper confirm that the EPTL has a significant abatement effect on $PM_{2.5}$ pollution and provides references for those seeking to reform China’s green tax system to achieve “carbon neutrality” and “carbon peak”. Additionally, the paper provides quantified evidence for countries around the world developing policies to control air pollution.

The results show that the AEEPTLPM has significant spatial heterogeneity, which is likely caused by differing EPTL rates, the efficiency of collection and administration, and so on. The EPTL stipulates that provincial governments can set local EPTL rates (according to their priorities) and that environmental protection tax revenues belong to the local government. However, the legislation is unclear on specific operation rules, and local governments are also unclear in practice, which leads to significant heterogeneity of the AEEPTLPM. Therefore, the conclusions of this study can provide an empirical basis for improving the EPTL. In addition, the environmental protection tax rate should be set scientifically. The tax rate standard is the price of pollution; only if it is set reasonably can polluter discharge behaviour be better regulated. Moreover, local governments should strengthen the collection and management of environmental protection taxes. Although the results show that five provinces (Henan, Anhui, Guangdong, Inner Mongolia, and Liaoning) are among the highest ten in PATIPME value, the AEEPTL on their industrial $PM_{2.5}$ emissions are not among the ten highest. This shows that the collection and administration of the environmental protection tax in these areas might not be strict enough, resulting in a serious loss of tax revenues. Therefore, the local administration of environmental taxes should be strengthened to improve the AEEPTL.

Based on measuring the AEEPTL on in-situ-monitored $PM_{2.5}$ concentrations and industrial $PM_{2.5}$ emissions, the SSI of the AEEPTLPM was established to quantise the spatial spillovers of the AEEPTLPM in mainland China. Previous studies have investigated the spatial spillovers of environmental regulations in China, for instance, in verifying the pollution shelter hypothesis [20,21]. Feng et al. [22] used the SDM to explore the spatial spillovers of environmental regulations in three urban agglomeration areas. However, researchers have not focused on the AEEPTLPM instead of the abatement effects of the synthetical environmental regulations. Furthermore, our study estimated the spatial spillover of each region by fully considering spatial heterogeneity. The results of the SSI calculated at the provincial level show that the AEEPTLPM has had significant spatial spillovers and that the degree and direction of spatial spillovers vary by region. This provides a new perspective for the regional coordination of atmospheric pollution control in China. Due to atmospheric circulation, local in-situ-monitored $PM_{2.5}$ concentrations might not coincide with local industrial $PM_{2.5}$ emissions. Thus, the existence of the spatial spillover of the AEEPTL indicates that regionally coordinated mechanisms should be considered in controlling atmospheric pollution, especially in assessing atmospheric pollution costs. In addition, if pollution increases in one region due to spatial spillover from neighbouring areas, the governments of neighbouring areas should use their environmental protection tax revenues to compensate for pollution spillover into other regions for pollution control.

## 5. Conclusions

Firstly, the EPTL has had significant abatement effects on in-situ-monitored $PM_{2.5}$ annual concentrations and the total annual emissions of industrial $PM_{2.5}$. Secondly, the AEEPTL on the monitored $PM_{2.5}$ concentrations and local industrial $PM_{2.5}$ emissions exhibit significant spatial heterogeneity. Thirdly, the AEEPTLPM in mainland China is correlated with the degree of $PM_{2.5}$ pollution. Specifically, the AEEPTL is better in areas with more serious levels of $PM_{2.5}$ pollution, and vice versa. Fourthly, the AEEPTLPM display significant spatial spillover characteristics, and SSI values differ by province. This study has the following three limitations. (1) The research period is relatively short. Researchers should continue to collect data and carry out long-term research. (2) Due to the availability of data, the $PM_{2.5}$ emissions data in 2018–2019 were obtained through estimation. Although the accuracy is high, obtaining actual survey data would be preferable. (3) The method estimating AEEPTLPM in this paper needs assumptions, it should be developed in the future study.

## Figures and Tables

**Table 1:** Relative errors and root mean square errors (RMSE) for annual total industrial $PM_{2.5}$ emissions in mainland China from 2013 to 2017.

Provincial Region	2013	2014	2015	2016	2017	RMSE
Beijing	−0.7%	2.9%	3.4%	−0.3%	−7.4%	3.9%
Tianjin	−5.5%	0.2%	12.6%	11.5%	−11.5%	9.5%
Hebei	−3.1%	4.4%	−2.7%	8.1%	−3.7%	4.8%
Shanxi	−6.5%	−0.3%	0.5%	1.9%	4.9%	3.8%
Inner Mongolia	−1.3%	1.2%	−2.6%	8.0%	0.7%	3.8%
Liaoning	−7.9%	−2.3%	8.6%	12.3%	1.6%	7.7%
Jilin	−10.5%	12.0%	6.0%	8.8%	−3.6%	8.7%
Heilongjiang	−1.7%	0.7%	3.6%	8.3%	−1.1%	4.1%
Shanghai	3.2%	2.3%	5.5%	3.6%	−7.3%	4.7%
Jiangsu	−1.8%	−6.5%	3.1%	5.0%	4.4%	4.5%
Zhejiang	−2.3%	−4.3%	−3.9%	10.9%	−0.4%	5.6%
Anhui	−2.4%	−2.0%	−1.1%	4.7%	0.3%	2.6%
Fujian	−1.2%	0.3%	−0.4%	2.6%	−0.6%	1.3%
Jiangxi	−3.7%	−0.9%	−1.5%	4.6%	3.8%	3.2%
Shandong	−1.0%	−0.7%	−1.4%	4.1%	1.5%	2.1%
Henan	0.7%	0.4%	−0.4%	3.3%	−2.8%	2.0%
Hubei	−4.1%	7.5%	−3.2%	5.6%	−1.4%	4.8%
Hunan	−3.7%	3.5%	3.1%	3.5%	−1.8%	3.2%
Guangdong	−2.3%	−0.2%	2.6%	1.1%	−1.0%	1.7%
Guangxi	−1.0%	−3.7%	−1.5%	9.4%	−1.2%	4.6%
Hainan	−9.5%	−4.3%	7.1%	−5.6%	7.3%	7.0%
Chongqing	−6.0%	−6.9%	−0.7%	6.9%	5.8%	5.8%
Sichuan	−5.5%	3.0%	6.8%	4.9%	−7.1%	5.7%
Guizhou	−4.8%	−0.2%	1.3%	−2.3%	6.2%	3.7%
Yunnan	−3.8%	4.0%	1.9%	3.2%	−2.9%	3.3%
Tibet	−0.3%	0.5%	0.4%	−4.3%	3.4%	2.5%
Shaanxi	−8.5%	−6.1%	−6.9%	8.4%	13.1%	9.0%
Gansu	−4.9%	2.5%	−1.9%	7.1%	2.0%	4.2%
Qinghai	−7.2%	1.6%	2.3%	0.1%	3.2%	3.8%
Ningxia	−3.8%	−3.0%	−4.3%	10.5%	−0.3%	5.5%
Xinjiang	−4.6%	0.8%	5.9%	2.6%	−5.3%	4.3%

**Table 2:** Spatial spillover index values of the abatement effects of the Environmental Protection Tax Law on $PM_{2.5}$ pollution in provincial Chinese regions.

Province	$E^{\left(GM\right)}$	$WE^{\left(GM\right)}$	$\frac{1}{\alpha }\cdot E^{\left(EI\right)}$	$\frac{1}{\beta }\cdot WE^{\left(EI\right)}$	$\frac{E^{\left(GM\right)}}{WE^{\left(GM\right)}}$	$\frac{\frac{1}{\alpha }\cdot E^{\left(EI\right)}}{\frac{1}{\beta }\cdot WE^{\left(EI\right)}}$	SSI
Beijing	3.05	2.56	139.99	703.26	1.19	0.20	5.98
Tianjin	2.71	2.48	173.29	1010.76	1.09	0.17	6.36
Hebei	2.40	1.96	1231.31	654.16	1.22	1.88	0.65
Shanxi	1.23	1.70	980.99	281.19	0.72	3.49	0.21
Inner Mongolia	1.38	1.61	8.74	285.82	0.86	0.03	28.11
Liaoning	1.59	1.74	365.22	505.25	0.91	0.72	1.26
Jilin	1.66	1.52	660.98	412.06	1.09	1.60	0.68
Heilongjiang	1.20	1.56	492.67	354.91	0.77	1.39	0.55
Shanghai	2.13	2.03	127.88	328.46	1.05	0.39	2.70
Jiangsu	2.02	2.08	472.65	442.61	0.97	1.07	0.91
Zhejiang	2.04	1.67	131.99	218.65	1.22	0.60	2.02
Anhui	1.92	1.96	308.94	477.40	0.98	0.65	1.51
Fujian	0.82	1.41	13.68	145.54	0.58	0.09	6.17
Jiangxi	1.52	1.64	17.22	437.64	0.93	0.04	23.63
Shandong	2.33	2.05	1003.52	498.06	1.14	2.01	0.57
Henan	2.18	1.82	357.92	467.68	1.20	0.77	1.57
Hubei	1.98	1.76	1047.31	344.77	1.12	3.04	0.37
Hunan	1.74	1.49	616.23	313.67	1.17	1.96	0.60
Guangdong	1.05	1.29	233.62	280.08	0.82	0.83	0.98
Guangxi	1.10	1.04	444.46	277.23	1.06	1.60	0.66
Hainan	0.80	1.16	-8.88	236.74	0.69	\	\
Chongqing	2.12	1.44	11.46	438.58	1.47	0.03	56.26
Sichuan	1.42	1.12	1027.05	174.14	1.27	5.90	0.22
Guizhou	1.25	1.09	375.24	319.19	1.14	1.18	0.97
Yunnan	0.38	0.88	437.78	213.38	0.43	2.05	0.21
Tibet	0.25	1.13	5.29	158.41	0.22	0.03	6.59
Shaanxi	1.29	1.62	73.09	244.37	0.80	0.30	2.67
Gansu	1.97	1.39	-77.44	181.36	1.41	\	\
Qinghai	1.25	1.23	32.24	200.43	1.02	0.16	6.34
Ningxia	1.50	1.54	79.02	251.67	0.97	0.31	3.09
Xinjiang	1.27	1.25	190.38	184.41	1.02	1.03	0.99

## Data Availability

The retrieved remote sensing data for $PM_{2.5}$ concentrations can be downloaded from https://sites.wustl.edu/acag/datasets/surface-pm2-5/ (accessed on 6 June 2021). The in-situ monitored $PM_{2.5}$ concentrations data can be obtained from https://air.cnemc.cn:18007/ (accessed on 6 June 2021). The $PM_{2.5}$ emission inventory data can be obtained from http://meicmodel.org/ (accessed on 6 June 2021).

## Supplementary Materials

The following supporting information can be downloaded at: https://www.mdpi.com/article/10.3390/ijerph19031440/s1, Figure S1: Spatial distribution of 1,497 in situ air quality monitoring stations in mainland China from 2015 to 2018; the coloured base map indicates the annual average $PM_{2.5}$ concentrations in mainland China in 2016.; Figure S2. Local trends of in-situ monitored $PM_{2.5}$ annual concentrations, the posterior median of the parameter, $k_{1i}^{\left(0\right)},$ estimated by the Bayesian space-time hierarchy model over the 31 provincial regions in China mainland from 2013 to 2017; Figure S3. Local trends of in-situ monitored $PM_{2.5}$ annual concentrations, the posterior median of the parameter, $k_{1i}^{\left(1\right)},$ estimated by the Bayesian space-time hierarchy model over the 31 provincial regions in China mainland from 2013 to 2019; Figure S4. Local trends of local total industrial PM_2.5 emissions, the posterior median of the parameter, $k_{1i}^{\left(0\right)},$ estimated by the Bayesian space-time hierarchy model over the 31 provincial regions in China mainland from 2013 to 2017; Figure S5. Local trends of local total industrial $PM_{2.5}$ emissions, the posterior median of the parameter, $k_{1i}^{\left(1\right)},$ estimated by the Bayesian space-time hierarchy model over the 31 provincial regions in China mainland from 2013 to 2019; Table S1: Summary of the number of prefectural regions covered by ground monitoring sites in 2013, 2014, and 2015–2018; Table S2: Validation of the accuracy of the remotely sensed annual $PM_{2.5}$ concentrations in 31 provincial regions.
