# Supplementary material for: Spatial Pattern and Spillover of Abatement Effect of Chinese Environmental Protection Tax Law on PM2.5 Pollution
Source: Int J Environ Res Public Health. 2022 Jan 27;19(3):1440. doi: 10.3390/ijerph19031440 (PMC8835502; doi:10.3390/ijerph19031440)
Supplement: Supplementary file 1 [file ijerph-19-01440-s001.zip › ijerph-1538604-supplementary.pdf]

Supplementary material

Table S1. Summary of the number of prefectural regions covered by ground monitoring sites in 2013, 2014, and 2015–2018

Provinces	Number of prefectural regions	Number of prefectural regions covered by monitoring sites in 2013	Number of prefectural regions covered by monitoring sites in 2014	Number of prefectural regions covered by monitoring sites in 2015–18
Beijing	18	12 (66.7%)	12 (66.7%)	18 (100%)
Tianjin	18	15 (83.3%)	15 (83.3%)	18 (100%)
Hebei	11	11 (100%)	11 (100%)	11 (100%)
Shanxi	11	1 (9.1%)	5 (45.5%)	11 (100%)
Inner Mongolia	12	1 (8.3%)	2 (16.7%)	12 (100%)
Liaoning	14	2 (14.3%)	6 (42.9%)	14 (100%)
Jilin	9	1 (11.1%)	1 (11.1%)	9 (100%)
Heilongjiang	13	1 (7.7%)	4 (30.8%)	13 (100%)
Shanghai	19	10 (52.6%)	10 (52.6%)	19 (100%)
Jiangsu	13	13 (100%)	13 (100%)	13 (100%)
Zhejiang	11	3 (27.3%)	3 (27.3%)	11 (100%)
Anhui	17	1 (5.9%)	3 (17.6%)	17 (100%)
Fujian	9	2 (22.2%)	2 (22.2%)	9 (100%)
Jiangxi	11	1 (9.1%)	2 (18.2%)	11 (100%)
Shandong	17	2 (11.8%)	2 (11.8%)	17 (100%)
Henan	17	1 (5.9%)	5 (29.4%)	17 (100%)
Hubei	13	1 (7.7%)	3 (23.1%)	13 (100%)
Hunan	14	1 (7.1%)	4 (28.6%)	14 (100%)
Guangdong	21	9 (42.9%)	9 (42.9%)	21 (100%)
Guangxi	14	1 (7.1%)	4 (28.6%)	14 (100%)
Hainan	3	1 (33.3%)	2 (66.7%)	3 (100%)
Chongqing	40	17 (42.5%)	17 (42.5%)	40 (100%)
Sichuan	21	1 (4.8%)	3 (14.3%)	21 (100%)
Guizhou	9	1 (11.1%)	2 (22.2%)	9 (100%)
Yunnan	16	1 (6.3%)	1 (6.3%)	16 (100%)
Tibet	7	1 (14.3%)	1 (14.3%)	7 (100%)
Shaanxi	10	1 (10.0%)	6 (60.0%)	10 (100%)
Gansu	14	1 (7.1%)	3 (21.4%)	14 (100%)
Qinghai	8	1 (12.5%)	1 (12.5%)	8 (100%)
Ningxia	5	1 (20.0%)	2 (40.0%)	5 (100%)
Xinjiang	14	1 (7.1%)	3 (21.4%)	14 (100%)

Table S2. Validation of the accuracy of the remotely sensed annual $PM_{2.5}$ concentrations in 31 provincial regions

Provinces	Error in 2015 ($\mu g/m^3$)	Relative error in 2015	Error in 2016 ($\mu g/m^3$)	Relative error in 2016
Beijing	3.53	4.6%	2.48	3.5%
Tianjin	–0.43	–0.6%	–2.01	–2.8%
Hebei	–0.53	–0.7%	–2.65	–3.7%
Shanxi	–0.45	–0.8%	–1.21	–2.0%
Inner Mongolia	1.65	4.2%	0.96	2.7%
Liaoning	–0.69	–1.2%	–0.33	–0.7%
Jilin	1.18	2.1%	1.10	2.6%
Heilongjiang	1.45	3.4%	0.91	2.7%
Shanghai	–0.05	–0.1%	0.33	0.7%
Jiangsu	–2.58	–4.3%	–1.03	–1.9%
Zhejiang	0.53	1.1%	1.60	3.7%
Anhui	–2.87	–4.8%	–0.77	–1.4%
Fujian	0.88	3.2%	1.21	4.8%
Jiangxi	–0.28	–0.7%	0.38	0.9%
Shandong	0.39	0.6%	–0.56	–0.9%
Henan	–0.58	–0.7%	–1.02	–1.4%
Hubei	1.48	2.3%	1.59	2.9%
Hunan	–0.86	–1.6%	–0.73	–1.5%
Guangdong	–0.70	–2.0%	0.54	1.7%
Guangxi	–0.55	–1.4%	0.26	0.7%
Hainan	–0.72	–3.5%	–0.65	–3.4%
Chongqing	–1.68	–3.0%	–1.84	–3.4%
Sichuan	–0.95	–1.9%	–0.14	–0.3%
Guizhou	–1.06	–2.9%	0.94	2.9%
Yunnan	1.03	4.5%	0.93	4.1%
Tibet	0.59	4.9%	0.14	1.1%
Shaanxi	2.42	4.7%	2.07	3.6%
Gansu	–1.28	–2.7%	–1.87	–4.1%
Qinghai	1.27	3.6%	1.33	4.3%
Ningxia	0.11	0.2%	0.59	1.4%
Xinjiang	–1.59	–2.9%	1.04	1.7%

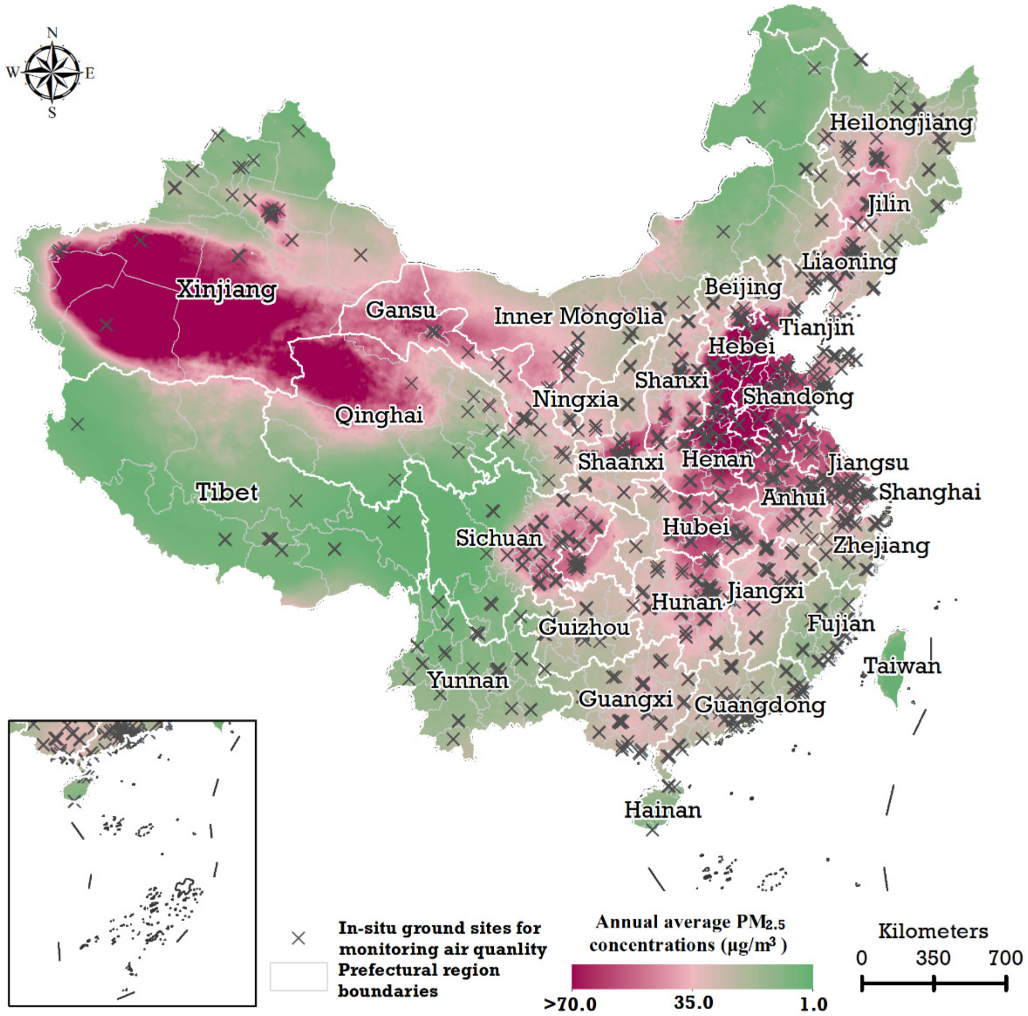

Figure S1. Spatial distribution of 1,497 in situ air quality monitoring stations in mainland China from 2015 to 2018; the coloured base map indicates the annual average $PM_{2.5}$ concentrations in mainland China in 2016.

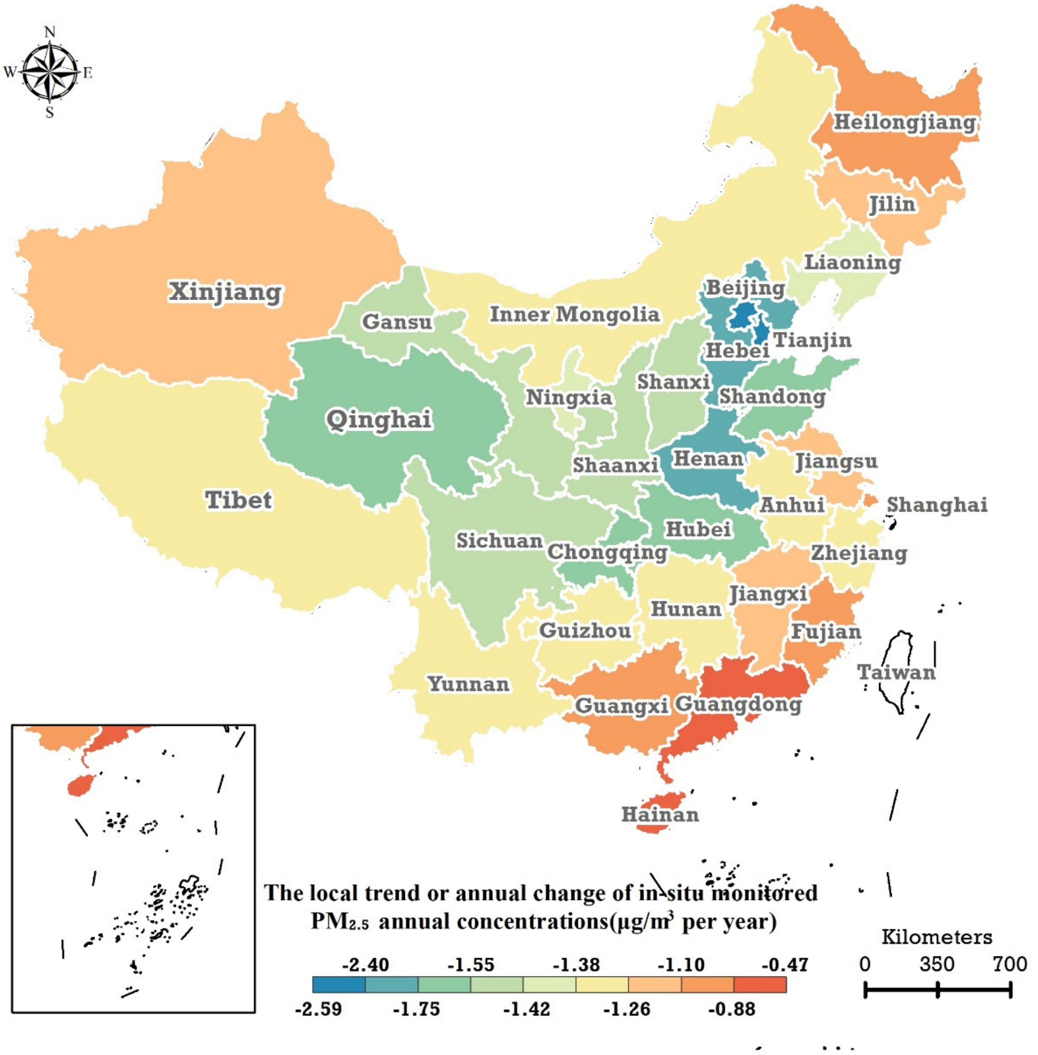

Figure S2. Local trends of in-situ monitored $PM_{2.5}$ annual concentrations, the posterior median of the parameter, $k_{1i}^{(0)}$, estimated by the Bayesian space-time hierarchy model over the 31 provincial regions in China mainland from 2013 to 2017

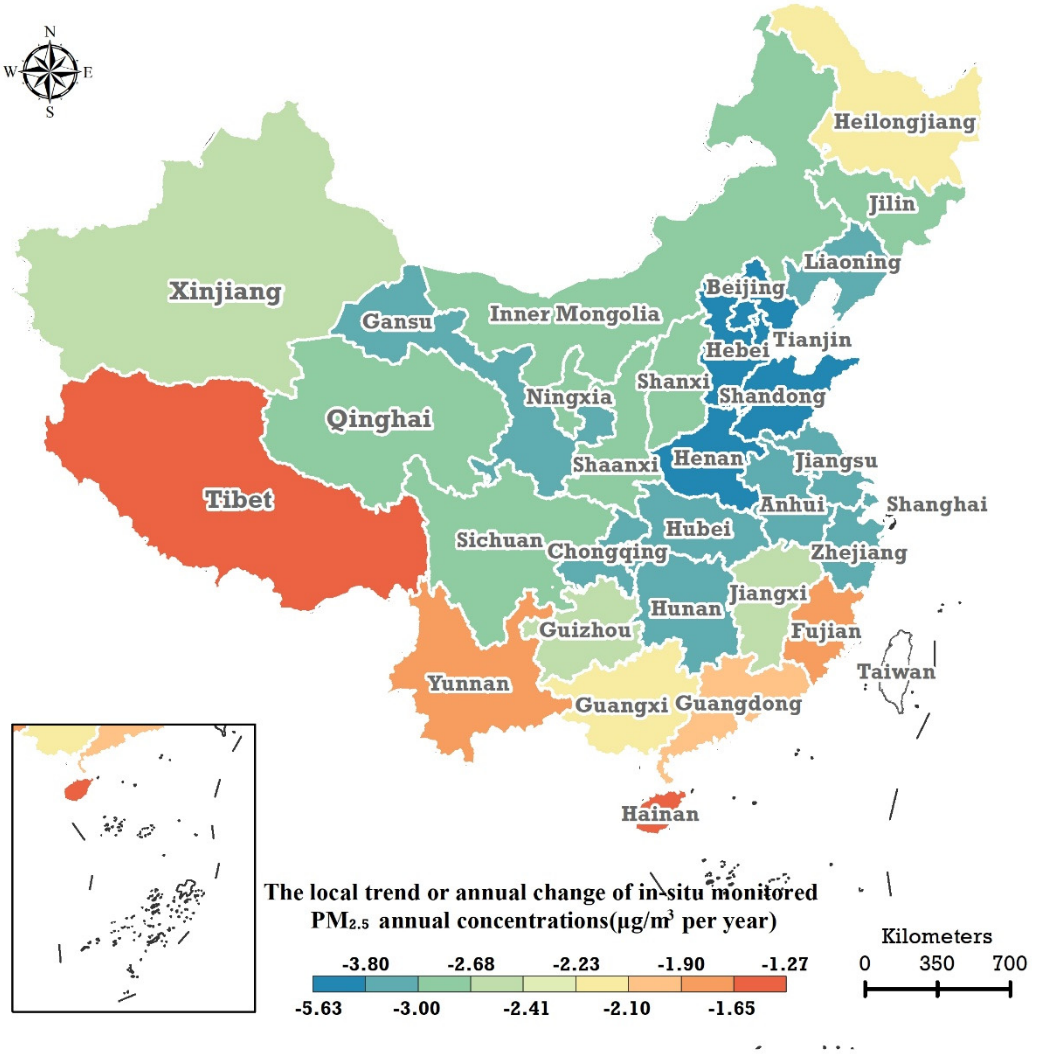

Figure S3. Local trends of in-situ monitored $PM_{2.5}$ annual concentrations, the posterior median of the parameter, $k_{1i}^{(1)}$, estimated by the Bayesian space-time hierarchy model over the 31 provincial regions in China mainland from 2013 to 2019

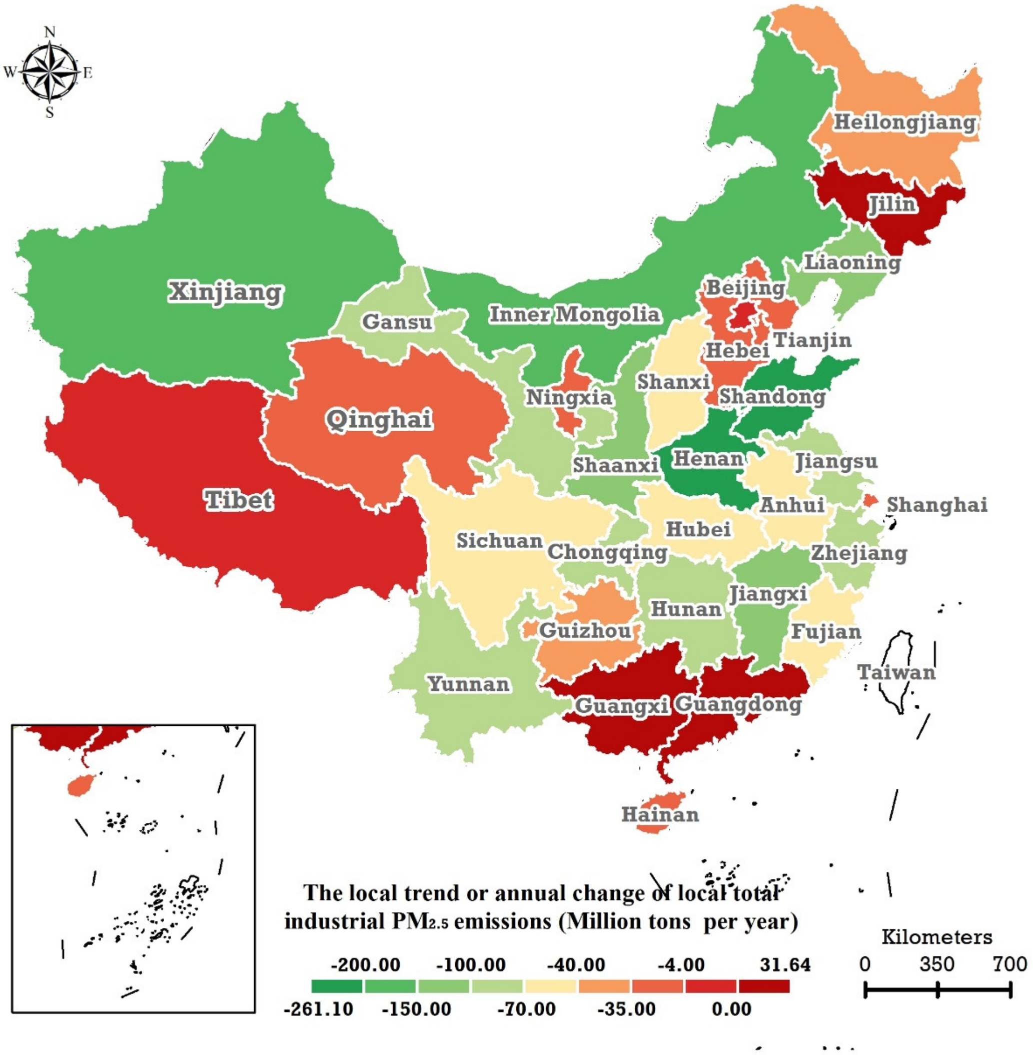

Figure S4. Local trends of local total industrial $PM_{2.5}$ emissions, the posterior median of the parameter, $k_{1i}^{(0)}$, estimated by the Bayesian space-time hierarchy model over the 31 provincial regions in China mainland from 2013 to 2017

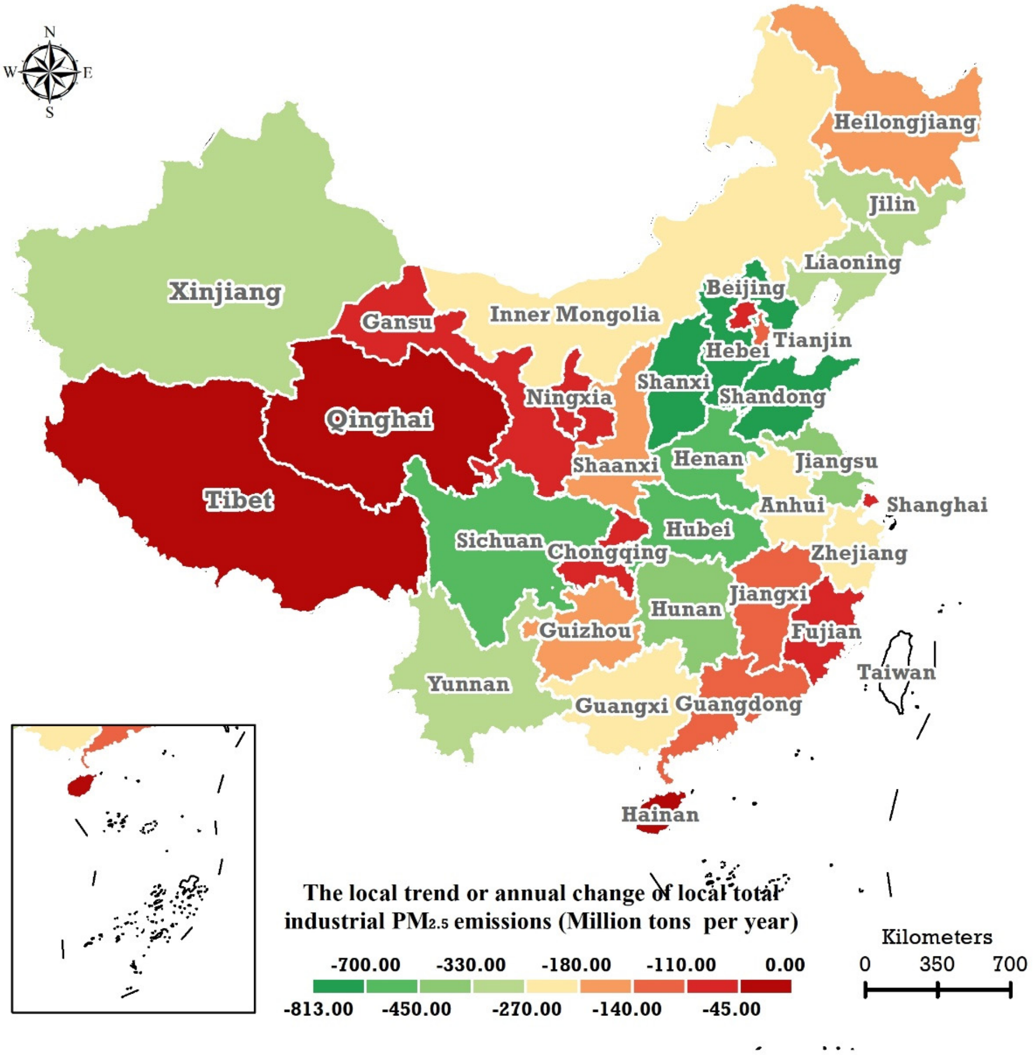

Figure S5. Local trends of local total industrial $PM_{2.5}$ emissions, the posterior median of the parameter, $k_{1i}^{(1)}$, estimated by the Bayesian space-time hierarchy model over the 31 provincial regions in China mainland from 2013 to 2019
